# Mutagenic Organized Recombination Process by Homologous In Vivo Grouping (MORPHING) for Directed Enzyme Evolution

**DOI:** 10.1371/journal.pone.0090919

**Published:** 2014-03-10

**Authors:** David Gonzalez-Perez, Patricia Molina-Espeja, Eva Garcia-Ruiz, Miguel Alcalde

**Affiliations:** 1 Departmento de Biocatálisis, Instituto de Catálisis y Petroleoquímica, Consejo Superior de Investigaciones Científicas (CSIC), Madrid, Spain; 2 Department of Chemical and Biomolecular Engineering, University of Illinois at Urbana-Champaign, Urbana, Illinois, United States of America; Instituto de Tecnologica Química e Biológica, UNL, Portugal

## Abstract

Approaches that depend on directed evolution require reliable methods to generate DNA diversity so that mutant libraries can focus on specific target regions. We took advantage of the high frequency of homologous DNA recombination in *Saccharomyces cerevisiae* to develop a strategy for domain mutagenesis aimed at introducing and *in vivo* recombining random mutations in defined segments of DNA. *M*utagenic *O*rganized *R*ecombination *P*rocess by *H*omologous *IN* vivo *G*rouping (MORPHING) is a *one-pot* random mutagenic method for short protein regions that harnesses the *in vivo* recombination apparatus of yeast. Using this approach, libraries can be prepared with different mutational loads in DNA segments of less than 30 amino acids so that they can be assembled into the remaining unaltered DNA regions *in vivo* with high fidelity. As a proof of concept, we present two eukaryotic-ligninolytic enzyme case studies: i) the enhancement of the oxidative stability of a H_2_O_2_-sensitive versatile peroxidase by independent evolution of three distinct protein segments (Leu28-Gly57, Leu149-Ala174 and Ile199-Leu268); and ii) the heterologous functional expression of an unspecific peroxygenase by exclusive evolution of its native 43-residue signal sequence.

## Introduction

In the past two decades directed evolution strategies have had a huge impact on protein engineering and synthetic biology [1]–[5]. Combining directed evolution with new computational and hybrid approaches has allowed researchers to design “smart” mutant libraries to address bottlenecks in enzyme functionality, helping to maintain the balance between activity and stability, or even creating novel catalytic activities [6], [7]. The use of non-adaptive evolution involving neutral genetic drift has been added to this arsenal of techniques to create polymorphic populations with the aim of enhancing protein robustness and substrate promiscuity [8]–[11].

Although the majority of the vast protein sequence space is probably non-functional, with 20^n^ possible permutations (if we exclude the introduction of non-natural amino acids), it is still far from being fully explored [12]. Advances in the field involve the use of ultrahigh-throughput screening methods and the construction of focused mutant libraries to restrict the sequence space [13], [14]. Most of the available methods used to create targeted libraries are based on computational studies that identify a limited number of positions for saturation mutagenesis, combinatorial and/or iterative [15]–[17]. A more recent approach is to introduce ancestral consensum mutations that have been identified by phylogenetic analysis and ancestral inference in extant enzymes [18]. Despite the wide array of focused evolution methods, there remains a need for consistent domain mutagenesis/recombination strategies targeting specific protein subsets for random mutagenesis and recombination, while conserving the remaining protein regions. Although this kind of focused-indiscriminate approach has received little attention in the literature [19], [20], it can effectively unmask structural determinants of specific enzymatic attributes, which can then be optimized using the aforementioned methods.

*Escherichia coli* is by far the most common host in directed evolution experiments of prokaryotic proteins. However, broad differences with eukaryotic cells (missing chaperones, different codon usage, lack of posttranslational modifications) preclude the use of this bacteria to engineer eukaryotic enzymes which mostly end up in misfolding and inclusion bodies formation. Alternatively, *Saccharomyces cerevisiae* is the model organism of choice for *in vitro* evolution of eukaryotic genes, permitting the development of comprehensive synthetic biology and metabolic engineering studies, particularly when dealing with the production of fuels and chemicals [21]–[24]. In recent years, a range of methods have been described in this yeast to construct mutant libraries with different mutational bias, to integrate multiple DNA fragments for creating combinatorial libraries or to assemble expression cassettes that generate fully autonomous artificial pathways [25]–[28]. The high frequency of homologous DNA recombination in *S. cerevisiae* permits to simply shuffle foreign genes creating multiple crossover events, to repair linearized vectors for *in vivo* cloning or to promote the molecular evolution of multigenic phenotypes [29]–[33].

Here, we present a simple, rapid and reliable random domain mutagenesis/recombination method for short fragments that is based on the physiological properties of *S. cerevisiae*. *M*utagenic *O*rganized *R*ecombination *P*rocess by *H*omologous *IN* vivo *G*rouping (MORPHING) randomly introduces mutations in specific protein segments using overlapping areas to favor *in vivo* splicing and recombination in yeast. The versatility of this method was evaluated in two case studies of ligninolytic oxidoreductases. First, we used MORPHING to enhance the oxidative stability of a versatile peroxidase (VP) from the basidiomycete *Pleurotus eryngii*. VP has three different catalytic sites for the oxidation of low-, medium- and high-redox potential compounds, which makes the enzyme extremely fragile in the presence of catalytic concentrations of H_2_O_2_ [34]. The second enzyme studied was the unspecific peroxygenase (UPO) from the edible mushroom *Agrocybe aegerita*, a heme-thiolate peroxidase with special catalytic capacity that includes oxygen transfer reactions [35]. Despite its importance in organic synthesis, heterologous functional expression and directed evolution of this enzyme has not yet been reported. We used MORPHING to exclusively target the native UPO signal peptide for evolution towards functional expression in yeast.

## Materials and Methods

VP from *Pleurotus eryngii* (the R4 mutant) was used as the parental type in the construction of the library. This R4 mutant was engineered for secretion by 4 rounds of directed evolution, resulting in expression levels of 22 mg/L [36]. The UPO1 variant (12C12) was generated by directed evolution in a previous study to functionally express the *upo1* gene (clone C1A-2) from *A. aegerita* [37] in *S. cerevisiae* (unpublished material). ABTS (2,2′-azino-bis(3-ethylbenzothiazoline-6-sulfonic acid)), *Taq* polymerase, and the *S. cerevisiae* transformation kit were purchased from Sigma-Aldrich (Madrid, Spain). The iProof High Fidelity DNA polymerase was purchased from Bio-Rad (USA). The Zymoprep Yeast Plasmid Miniprep Kit and Zymoclean Gel DNA Recovery Kit were obtained from Zymo Research (Orange, CA, USA), while NBD (5-nitro-1,3-benzodioxole) was purchased from TCI America (USA). The *Escherichia coli* XL2-Blue competent cells and the GeneMorph II Kit (mutazyme II polymerase) were from Stratagene (La Jolla, CA, USA), and the uracil independent and ampicillin resistance pJRoC30 shuttle vector was obtained from the California Institute of Technology (CALTECH, USA). The protease-deficient *S. cerevisiae* strain BJ5465 (*α ura3-52 trp1 leu2Δ1 his3Δ200 pep4::HIS3 prb1Δ1.6R can1 GAL*) was obtained from LGCPromochem (Barcelona, Spain), the NucleoSpin Plasmid kit was purchased from Macherey-Nagel (Germany), and the restriction enzymes BamHI and XhoI from New England Biolabs (Hertfordshire, UK). All chemicals were of reagent-grade purity.

### Culture media

Minimal medium (SC) contained 0.67% (w/v) sterile yeast nitrogen base, 1.92 g/L sterile yeast synthetic drop-out medium supplement without uracil, 2% (w/v) sterile D-raffinose and 25 µg/mL chloramphenicol. YP medium contained 10 g yeast extract, 20 g peptone and *dd*H_2_O to 650 mL. Flask expression medium contained 720 mL YP, 67 mL 1 M KH_2_PO_4_ buffer (pH 6.0), 111 mL 20% (w/v) D-galactose, 25 g/L ethanol, 500 or 300 mg/L bovine hemoglobin (for VP and UPO, respectively), 1 mM CaCl_2_ for VP or 2 mM MgSO_4_ for UPO, 1 mL 25 g/L chloramphenicol and *dd*H_2_O to 1000 mL. Microplate expression medium contained 720 mL YP, 67 mL 1 M KH_2_PO_4_ buffer (pH 6.0), 111 mL 20% (w/v) D-galactose, 100 or 50 mg/L bovine hemoglobin (for VP and UPO, respectively), 2 mM MgSO_4_ for UPO, 1 ml 25 g/L chloramphenicol and *dd*H_2_O to 1000 mL. YPD solution contained 1% (w/v) yeast extract, 2% (w/v) peptone, 2% (w/v) sterile D-glucose and 25 µg/mL chloramphenicol. SC drop-out plates contained 0.67% (w/v) sterile yeast nitrogen base, 1.92 g/L (w/v) sterile yeast synthetic drop-out medium supplement without uracil, 2% (w/v) bacto agar, 2% (w/v) sterile D-glucose and 25 µg/mL chloramphenicol. Luria-Bertani (LB) medium was prepared with 1% (w/v) peptone, 0.5% (w/v) yeast extract, 1% (w/v) NaCl and 100 µg/mL ampicillin.

### MORPHING Protocol

All the PCR products generated were cleaned, concentrated and loaded onto a low melting-point preparative agarose gel and purified using the Zymoclean Gel DNA Recovery Kit (Zymo Research). The PCR products were cloned by replacing the corresponding parental gene in pJRoC30. To remove the parental gene, the plasmid was linearized with BamHI and XhoI. The pJRoC30-R4 variant was used as a template to construct MORPHING libraries of VP, while the pJRoC30-12C12 was used as the template to construct MORPHING libraries of UPO signal peptide.

For VP MORPHING, the whole gene was fragmented into three different segments in individual PCR reactions. Each segment contained homologous overhangs of ∼50 bp that overlapped one another to promote *in vivo* cloning in yeast. The targeted regions were subjected to random mutagenesis while the remaining segments were amplified by high-fidelity polymerases (**Figure S1**). The distal His environment region was selected to tune mutational loads. Small mutant libraries of around 500 clones were screened to optimize the mutagenic conditions. A similar protocol was followed for UPO MORPHING but in this case the UPO gene was split into two segments, one containing the signal peptide and the other the mature protein.

#### 1. VP MORPHING

i) Mutagenic PCR of targeted regions: Reaction mixtures were prepared in a final volume of 50 µL containing the following: DNA template (0.1 ng/µL or 0.92 ng/µL), 90 nM Forward primer, 90 nM Reverse primer (different forward (F) and reverse (R) primers were used to amplify each region; **Table S1, Figure S1**), 0.3 mM dNTPs (0.075 mM each), 3% (v/v) dimethylsulfoxide (DMSO), 1.5 mM MgCl_2_, increasing concentrations of MnCl_2_ (0.01, 0.05, 0.1, 0.2, 0.4 mM) and 0.05 U/µL *Taq* polymerase. Error prone-PCRs were carried out on a gradient thermocycler (Mycycler, BioRad, USA) using the following conditions: 95°C for 2 min (1 cycle); 94°C for 45 s, 56°C for 45 s, 74°C for 45 s (28 cycles); and 74°C for 10 min (1 cycle). For the proximal His environment and Met environment, the final DNA template concentration was 0.92 ng/µL and the MnCl_2_ concentration was set at 0.2 mM and 0.01 mM, independently for both targeted regions.

ii) High-fidelity PCR in non-mutated segments: Reaction mixtures were prepared in a final volume of 50 µL containing: DNA template (0.2 ng/µL), 0.5 µM Forward primer, 0.5 µM Reverse, (different forward (F) and reverse (R) primers were used to amplify each region; **Table S1, Figure S1**), 0.8 mM dNTPs (0.2 mM each), 3% (v/v) dimethylsulfoxide (DMSO), and 0.02 U/µL iProof polymerase. High fidelity PCRs were performed on a gradient thermocycler using the following conditions: 98°C for 30 s (1 cycle); 98°C for 10 s, 45 or 50°C for 30 s, 72°C for 1 min (28 cycles); and 72°C for 10 min (1 cycle).

iii) Reassembly of the whole gene: The whole gene was reassembled *in vivo* and recombined by transformation into *S. cerevisiae* cells using the Yeast Transformation Kit. The DNA transformation mixture contained the linearized plasmid (200 ng) mixed with the targeted region, as well as the segments upstream and downstream of those regions (400 ng per segment). Transformed cells were plated on SC drop-out plates and incubated for 3 days at 30°C. Subsequently, the mutant libraries were subjected to the HTP-protocol for oxidative stability, as described below.

#### 2. UPO MORPHING

i) Mutagenic PCR in signal peptide: Reaction mixtures were prepared in a final volume of 50 µL containing the following: DNA template (0.92 ng/µL), 90 nM RMLN F primer, 90 nM MORPH SP R primer (**Table S1**), 0.3 mM dNTPs (0.075 mM each), 3% (v/v) dimethylsulfoxide (DMSO), 1.5 mM MgCl_2_, 0.1 mM MnCl_2_ and 0.05 U/µL *Taq* polymerase. Error-prone PCR was carried out on a gradient thermocycler using the following parameters: 95°C for 2 min (1 cycle); 94°C for 45 s, 50°C for 45 s, 74°C for 30 s (28 cycles); and 74°C for 10 min (1 cycle).

ii) High-fidelity PCR of non-mutated segment: Reaction mixtures were prepared in a final volume of 50 µL containing DNA template (0.2 ng/µL), 0.5 µM MORPH SP D primer, 0.5 µM RMLC R primer (**Table S1**), 1 mM dNTPs (0.25 mM each), 3% (v/v) dimethylsulfoxide (DMSO), and 0.02 U/µL iProof polymerase. High fidelity PCR was carried out on the gradient thermocycler using the following conditions: 98°C for 30 s (1 cycle); 98°C for 10 s, 55°C for 25 s, 72°C for 45 s (28 cycles); and 72°C for 10 min (1 cycle).

iii) Reassembly of the whole gene: The whole gene was reassembled *in vivo* and recombined by transformation into *S. cerevisiae* cells using the Yeast Transformation Kit. The DNA transformation mixture contained the linearized plasmid (100 ng) mixed with the targeted region, as well as the mature protein (200 ng per segment). Transformed cells were plated on SC drop-out plates and incubated for 3 days at 30°C. Subsequently, the mutant libraries were subjected to the HTP-protocol to assess activity as described below.

### Directed evolution of whole UPO gene

The whole UPO gene including its signal peptide was subjected to one round of directed evolution by *In vivo* Assembly of Mutant libraries (IvAM) [27].

#### 1. Mutagenic PCR with *Taq* polymerase

Reaction mixtures were prepared in a final volume of 50 µL containing: DNA template (0.1 ng/µL), 90 nM RMLN F primer, 90 nM RMLC R primer (**Table S1**), 0.3 mM dNTPs (0.075 mM each), 3% (v/v) dimethylsulfoxide (DMSO), 1.5 mM MgCl_2_, 0.01 mM MnCl_2_ and 0.05 U/µL *Taq* polymerase. Error-prone PCR was carried out under the following conditions: 95°C for 2 min (1 cycle); 94°C for 45 s, 53°C for 45 s, 74°C for 3 min (28 cycles); and 74°C for 10 min (1 cycle).

#### 2. Mutagenic PCR with Mutazyme II

Reaction mixtures were prepared in a final volume of 50 µL containing: DNA template (56 ng/µL), 0.37 µM RMLN F primer, 0.37 µM RMLC R primer (**Table S1**), 0.8 mM dNTPs (0.2 mM each), 3% (v/v) dimethylsulfoxide (DMSO), and 0.05 U/µL Mutazyme II. Error-prone PCR was carried out using the following parameters: 95°C for 2 min (1 cycle); 94°C for 45 s, 53°C for 45 s, 74°C for 3 min (28 cycles); and 74°C for 10 min (1 cycle).

#### 3. In vivo recombination of mutant libraries in S. cerevisiae

*Taq* polymerase library and Mutazyme library were added in equimolar concentrations (200 ng each) to the linearized vector (100 ng). Transformed cells were plated on SC drop-out plates and incubated for 3 days at 30°C. Thereafter, the mutant libraries were subjected to the HTP-protocol to assay activity as described below.

### Construction of the evolved signal peptide F12Y-A14V-R15G-A21D by site-directed mutagenesis and fusion to the native *upo* gene

#### 1. Construction of the evolved signal peptide (SP*) by site-directed mutagenesis

Reaction mixture was prepared in a final volume of 50 µL containing: DNA template (0.2 ng/µL), 0.5 µM Forward primer RMLN F, 0.5 µM Reverse primer SP* R (**Table S1**), 1 mM dNTPs (0.25 mM each), 3% (v/v) dimethylsulfoxide (DMSO), and 0.02 U/µL iProof polymerase. High fidelity PCR1 was carried out on the gradient thermocycler under the following conditions: 98°C for 30 s (1 cycle); 98°C for 10 s, 47°C for 25 s, 72°C for 15 s (28 cycles); and 72°C for 10 min (1 cycle).

#### 2. Construction of fusion gene (evolved signal peptide plus the native upo) by In Vivo Overlap Extension (IVOE)

the native *upo* was amplified by high-fidelity PCR in a final volume of 50 µL containing: DNA template (0.2 ng/µL), 0.5 µM Forward primer SP* F, 0.5 µM Reverse primer RMLC R (**Table S1**), 1 mM dNTPs (0.25 mM each), 3% (v/v) dimethylsulfoxide (DMSO), and 0.02 U/µL iProof polymerase. High fidelity PCR was carried out on the gradient thermocycler under the following conditions: 98°C for 30 s (1 cycle); 98°C for 10 s, 52°C for 25 s, 72°C for 40 s (28 cycles); and 72°C for 15 min (1 cycle). PCR fragments corresponding to the SP* and the native *upo* (200 ng each) were recombined together with the linearized vector (100 ng) by IVOE [25].

### Combinatorial saturation mutagenesis experiments in VP

Reaction mixtures were prepared in a final volume of 50 µL containing: DNA template (0.2 ng/µL), 0.2 µM Forward primer, 0.2 µM Reverse primer (RMLN F/MET SAT R primers and MET SAT F/RMLC R for first PCR and second PCR, respectively, (**Table S1**)), 0.8 mM dNTPs (0.2 mM each), 3% (v/v) dimethylsulfoxide (DMSO), and 0.02 U/µL iProof polymerase. High fidelity PCRs were carried out on the gradient thermocycler under the following conditions: 98°C for 30 s (1 cycle); 98°C for 10 s, 53°C for 30 s, 72°C for 1 min (28 cycles); and 72°C for 10 min (1 cycle). The whole gene was reassembled *in vivo* and recombined by transformation into *S. cerevisiae* cells using the Yeast Transformation Kit. The DNA transformation mixture was composed of the linearized plasmid (200 ng) mixed with the mutated fragments (400 ng per fragment). Transformed cells were plated on SC drop-out plates and incubated for 3 days at 30°C. Thereafter, the mutant libraries were subjected to the HTP-protocol described below.

### High-throughput oxidative stability assay of VP

Individual clones were selected and cultured in sterile 96-well plates (Greiner Bio-One GmbH, Germany) containing 50 µL per well of SC minimal medium. In each plate, column number 6 was inoculated with the parental R4 mutant as an internal standard, and well-H1 (containing minimal medium supplemented with uracil) was inoculated with untransformed *S. cerevisiae* as a negative control. Plates were wrapped in parafilm to prevent evaporation and incubated at 30°C, 225 rpm and 80% relative humidity in a humidity shaker (Minitron-INFORS, Biogen, Spain). After 48 h, 160 µL of expression medium was added to each well and the plates were incubated for a further 24 h. The plates (master plates) were centrifuged for 15 min at 3000 rpm and 4°C (Eppendorf 5810R centrifuge, Germany) and the master plates were duplicated with the help of a robot (Liquid Handler Quadra 96–320, Tomtec, Hamden, CT, USA) by transferring 20 µL of supernatant into two replica plates: the initial activity plate (IA plate) and the residual activity plate (RA plate). Next, 180 µL of stability buffer (20 mM sodium tartrate buffer, pH 5.0: Buffer A) was added to the IA plates and 180 µL of incubation solution (Buffer A containing 0.3 mM H_2_O_2_) was added to the RA plates using a Multidrop robot (Multidrop Combi, ThermoFischer Scientific, Vantaa, Finland). Both plates were briefly stirred and incubated at room temperature for 60 min, such that the activity assessed in the RA plates was reduced by 2/3rds with respect to the initial activity of the parental type. The supernatants (20 µL) were transferred from both RA and IA plates to new plates to measure the residual and initial activity values by adding ABTS in specific buffers: 180 µL of 100 mM sodium tartrate buffer [pH 3.5] containing 2 mM ABTS and 0.1 mM H_2_O_2_ to estimate of residual activity; and 180 µL of 100 mM sodium tartrate buffer [pH 3.5] containing 2 mM ABTS and 0.13 mM H_2_O_2_ to estimate the initial activity. The plates were stirred briefly and the absorption at 418 nm (ε_ABTS_^•+^ = 36,000 M^−1^ cm^−1^) was recorded (end-point mode, t_0_) on a plate reader (SPECTRAMax Plus 384, Molecular Devices, Sunnyvale, CA). The plates were then incubated at room temperature until a green color developed and the absorption was measured again (t_1_). The relative activities were calculated from the difference between the absorption value after incubation and that of the initial measurement normalized to the parental type in the corresponding plate (Δt_1_-t_0_). Oxidative stability values were calculated as the ratio between residual activity and the initial activity values (RA/IA). To rule out false positives, two consecutive re-screenings were carried out. Moreover, a third re-screening was performed to determine the increase in the apparent half-life of each selected variant (*t*_1/2_ H_2_O_2_, expressed in minutes) relative to the parental R4 in different molar ratios [H_2_O_2_]/[VP in supernatants].

First re-screening: Aliquots of 5 µL of the best clones were removed from the master plates and used to inoculate 50 µL of minimal medium in new 96-well plates. Columns 1 and 12, and rows A and H, were not used to prevent the appearance of false positives. After incubating for 24 h at 30°C, 225 rpm, and 80% relative humidity, 5 µL was transferred to the adjacent wells and incubated for a further 24 h. Finally, 160 µL of expression medium was added and the plates were incubated for another 24 h. Accordingly, each mutant was grown in 4 wells. The parental types were subjected to the same procedure (row D, wells 7–11) and the plates were assessed using the same protocols as those used for the screening described above.

Second re-screening: An aliquot from the wells with the best clones in the first re-screening was inoculated in 3 mL of YPD and incubated at 30°C and 225 rpm for 24 h, recovering the plasmids from these cultures (Zymoprep Yeast Plasmid Miniprep Kit). As the product of the zymoprep was very impure and the concentration of DNA extracted very low, the zymoprep mixtures containing shuttle vectors were transformed into super-competent *E. coli* cells (XL2-Blue, Stratagene) and plated on LB/amp plates. Single colonies were picked and used to inoculate 5 mL LB/amp media, and they were grown overnight at 37°C and 225 rpm. The plasmids were then extracted (NucleoSpin Plasmid kit, Macherey-Nagel, Germany) and *S. cerevisiae* was transformed with plasmids from the best mutants as well as with the parental type. Five colonies for each mutant were selected and re-screened as described above.

Third re-screening (determination of *t*_1/2_ H_2_O_2_): A single colony from the *S. cerevisiae* clone containing the parental R4, the new mutants and untransformed yeast were picked from a SC drop-out plate (SC supplemented with uracil for untransformed cells), used to inoculate 5 mL of minimal medium, and incubated for 48 h at 30°C and 225 rpm (Minitron-INFORS, Biogen, Spain). An aliquot of cells was removed and used to inoculate a final volume of 5 mL of minimal medium in a 50 mL falcon tube (optical density, OD_600_ = 0.3), and they were incubated until two growth phases had been completed (6–8 h, OD_600_ = 1). Thereafter, 9 mL of expression medium (500 mg/L bovine hemoglobin) was inoculated with 1 mL of this preculture in a 100 mL flask (OD_600_ = 0.1). After incubating for ∼48 h at 30°C and 225 rpm (maximal VP activity; OD_600_ = 25–30), the cells were separated by centrifugation for 15 min at 3000 rpm and 4°C (Eppendorf 5810R Centrifuge, Germany), and the supernatants were collected and stored at 4°C. The protein concentration was estimated from supernatants using the Bio-Rad protein assay kit, (Bio-Rad, USA). VP apparent concentration was calculated as the difference between the total protein content of yeast expressing VP and that in its absence (from non-transformed yeast cells -lacking VP gene-). The parental R4 and the mutants (final concentration 0.1 µM) were incubated at room temperature in 20 mM sodium tartrate buffer [pH 5.0] in the presence or absence of different concentrations of H_2_O_2_ (0.3, 0.6 mM). Aliquots (20 µL) were removed at different times and the residual/initial activities were measured in a final volume of 200 µL containing 100 mM sodium tartrate buffer [pH 3.5], 2 mM ABTS and 0.13 mM or 0.16 mM H_2_O_2_ (for samples incubated in the presence of 0.3 and 0.6 mM H_2_O_2_, respectively). The residual activity was determined relative to the corresponding mutant incubated in the absence of H_2_O_2_, taking into account the final concentration of H_2_O_2_ in each activity assay. Each point represents the mean of three independent experiments performed in triplicate. Increment in apparent half-lives (Δ*t*_1/2_ *vs* H_2_O_2_) were calculated for each mutant at given molar ratio VP (supernatants) : H_2_O_2_.

### High-throughput screening assay for UPO secretion

Individual clones were selected and inoculated in sterile 96-well plates (Greiner Bio-One GmbH, Germany) containing 50 µL per well of SC minimal medium. In each plate, column number 6 was inoculated with the corresponding parental type, and one well (H1-control) was inoculated with untransformed *S. cerevisiae* cells in minimal medium containing uracil. The plates were wrapped in parafilm to prevent evaporation and incubated at 30°C, 225 rpm and 80% relative humidity in a humidity shaker. After 48 h, 160 µL of expression medium was added to each well and the plates were incubated for 48 h. The plates (master plates) were centrifuged (Eppendorf 5810R Centrifuge, Germany) for 15 min at 3000 rpm and 4°C, and the supernatants (20 µL) were transferred from the master plate to two replica plates by a robot (Liquid Handler Quadra 96–320, Tomtec, Hamden, CT, USA), adding the reaction mixture (180 µL) together with ABTS or NBD to each replica plate. Both colorimetric assays were used for properly detecting secretion levels improvements regardless of the substrate used (ABTS is typically used for assessing peroxidative activity whereas NBD for peroxygenase activity). The reaction mixture with ABTS contained 100 mM sodium citrate/phosphate buffer [pH 4.4], 0.3 mM ABTS and 2 mM H_2_O_2_, while the reaction mixture with NBD contained 100 mM potassium phosphate buffer [pH 7.0], 1 mM NBD, 15% (v/v) acetonitrile and 1 mM H_2_O_2_. Plates were stirred briefly and the initial absorptions at 418 nm (ε_ABTS_^•+^ = 36,000 M^−1^ cm^−1^) and 425 nm (ε_NBD_ = 9,700 M^−1^ cm^−1^) were recorded on the plate reader (SPECTRAMax Plus 384, Molecular Devices, Sunnyvale, CA). The plates were then incubated at room temperature until a green (ABTS) or yellow (NBD) color developed, and the absorption was measured again. The values were normalized against the parental type in the corresponding plate. To rule out false positives, two re-screenings were carried out as described above for VP.

### Thermostability assay (T_50_)

Appropriate dilutions of the supernatants were prepared such that aliquots (20 µL) produced a linear response in kinetic mode. A gradient profile was constructed using a thermocycler (Mycycler, Bio-Rad, USA) for the selected mutants and the parental type, using 50 µL for each point in a gradient scale ranging from 30 to 80°C. After a 10 min incubation, samples were removed and chilled on ice for 10 min. Thereafter, 20 µL samples were removed and incubated for 5 min at room temperature. Finally, 180 µL of 100 mM sodium tartrate buffer [pH 3.5], 2 mM ABTS and 0.1 mM H_2_O_2_ was added to the samples to measure activities. The thermostability values were calculated as the ratio between the residual activity at different temperature points and the initial activity at room temperature. The T_50_ value was determined as the transition midpoint of the inactivation curve of the protein as a function of temperature, which in our case was defined as the temperature at which the enzyme lost 50% of its initial activity after a 10 min incubation.

### DNA sequencing

Plasmids containing the VP/UPO variants were sequenced on an ABI 3730 DNA Analyzer-Applied Biosystems Automatic Sequencer at the Secugen (CIB, Madrid). The following primers were designed using Fast-PCR software (University of Helsinki, Finland):

for VP: RMLN F; 3R-direct (5′-GTTCCATCATCGCGTTCG-3′); 5F-reverse (5′-GGATTCCTTTCTTCTTGG-3′) and RMLC R (**Table S1**). For UPO: RMLN F; upo1 sec direct (5′-GAAGGCGACGCCAGTATGACC-3′); upo1 sec reverse (5′-GGTCATACTGGCGTCGCCTTC-3′) and RMLC R (**Table S1**).

### Protein and homology modeling

The crystal structure of VPL2 from *P. eryngii* at 2.8 Å resolution (1 Å = 0.1 nm, PDB ID: 3FJW) was used to generate a model to map the new mutations found with the help of the PyMOL Molecular Visualization System (Schrödinger). A homology model was generated by carrying out a structural alignment in PyMOL with the following crystal structures (PDB IDs are indicated): 3FJW, native VP from *P. eryngii*; 1IYN, recombinant chloroplastic ascorbate peroxidase (ApX) from *N. tabacum* expressed in *E. coli*; 3M5Q, native manganese peroxidase (MnP) isozyme 1 from *P. chrysosporium*; 1H3J, native peroxidase from *C. cinerea* (CiP); and 1W4W, recombinant horseradish peroxidase C1A from horseradish (HRP) expressed in *E. coli*. IDENTITY and SIMILARITY percentages were obtained using SEQUENCE SIMILARITY AND IDENTITY Software (http://imed.med.ucm.es/Tools/sias.html).

## Results and Discussion

**Figure 1:**
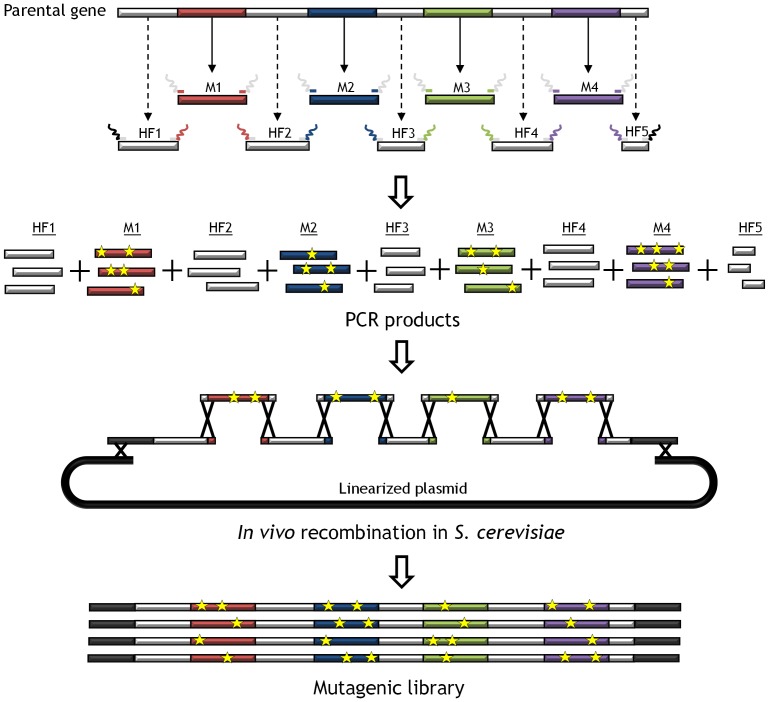
General approach for MORPHING. Segments subjected to random mutagenesis (M1 to M4) are shown in color and the non-mutagenized high fidelity amplified segments (HF1 to HF5) in light grey. After PCR, the pool of segments is co-transformed into *S. cerevisiae* along with the linearized vector. Overlapping areas of ∼50 bp flanking each segment allow specific crossover events to occur between fragments (represented by crosses), giving rise to an autonomously repaired vector carrying a full version of the target gene with random mutations (yellow stars) only in the defined regions.

MORPHING is a method of generating DNA diversity based on the high frequency of homologous recombination of *S. cerevisiae*. In a single step, this approach allows us to assemble delimited randomly mutagenized regions with the remaining, unaltered fragments of a gene. Unlike most evolution methods focused in restricted areas, mutations are randomly generated and they do not depend on the engineering of a set of spiked/degenerate synthetic oligonucleotides. By MORPHING, small segments are targeted and subjected to error-prone PCR with defined mutational frequencies, while the remaining portions of the gene are amplified using high-fidelity polymerases (**Figure 1**). Error-prone PCR methods have the drawback of codon bias although they can be modified by alternating between different polymerases in successive generations of evolution. Indeed, standard *Taq* polymerases (with a transition/transversion ratio [T_s_/T_v_] ranging from 2.9 to 0.8, [38]) were employed in our mutagenic experiments, although mutational bias may be altered by combining this protocol with other well-known polymerases and mutational strategies [27], [36], [39]. The pool of mutated/conserved fragments and the linearized plasmid are subjected to *one-pot* repair and cloned *in vivo*, giving rise to a complete autonomously replicating plasmid upon transformation in yeast, without the need for additional PCR reactions or ligation/amplification steps. The number of crossover events (*n*+1, where *n* is the number of fragments) between segments is directly proportional to the number of segments assembled, allowing several regions to be studied alone or in a combinatorial manner. Depending on the distance between mutations, crossover events can occur between the different mutations in the target fragment(s), mediated by the *S. cerevisiae* recombination machinery, fostering enrichment. The success of this method is facilitated by the high fidelity DNA splicing of fragments through the small overhangs with overlapping sequences of ∼50 bp that flank each segment. These overhangs ensure the *in vivo* reconstitution of the whole gene with random mutations only in the segments specifically targeted. Under these rules, up to six recombination events between fragments can be created without significantly affecting transformation efficiencies (∼10^5^ clones per transformation reaction can be obtained, which are good enough to screen mutant libraries).

### Engineering oxidative stability

**Figure 2:**
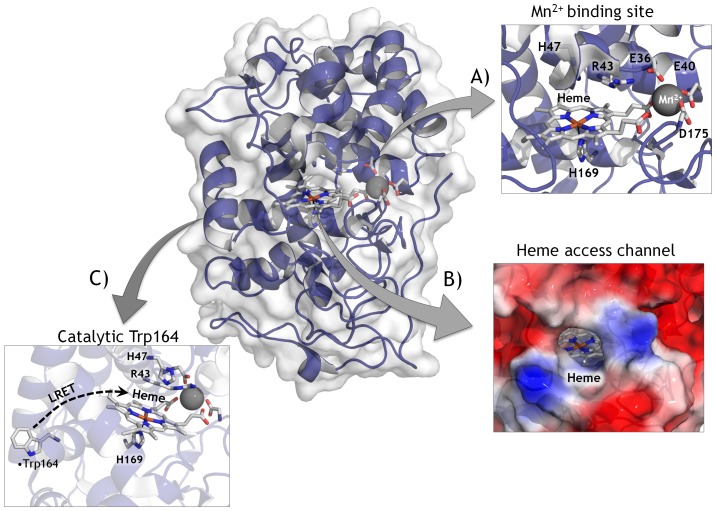
Overview of Versatile Peroxidase from *Pleurotus eryngii* (PDB ID: 3FJW). Heme prostetic group and catalytic residues are shown as sticks in CPK color. The three catalytic sites are shown in detail. (A) The Mn^2+^ binding site (Mn^2+^, black sphere), the distal and proximal His (His47 and His169) the Arg43 involved in H_2_O_2_ reduction and the coordinating triad (Glu36, Glu40, Asp175) are depicted. (B) Heme access channel represented as electrostatics surface with heme group (stick mode) at the bottom of channel. (C) Catalytic Trp164 (LRET; long range electron transfer).

We first used this protocol to engineer oxidative stability in an evolved VP variant, the VP-R4 mutant generated in a previous directed evolution experiment to enhance its functional expression in yeast and its stability [36]. The sensitivity of VP (EC 1.11.1.16) to peroxides is the highest reported for any peroxidase to date being strongly inhibited in the presence of catalytic concentrations of H_2_O_2_ due to a mechanism-based phenomenon known as suicide inactivation that is common to all peroxidases [40]. The inherent fragility of VP is explained by its complex structure. With a redox potential of over +1.2 V, three different catalytic sites (the heme domain, a catalytic tryptophan located at the protein's surface and the Mn^2+^ binding site), and two access channels, the dense production and traffic of free radicals jeopardizes the enzyme's stability and function, (**Figure 2**) [34], [36].

**Figure 3:**
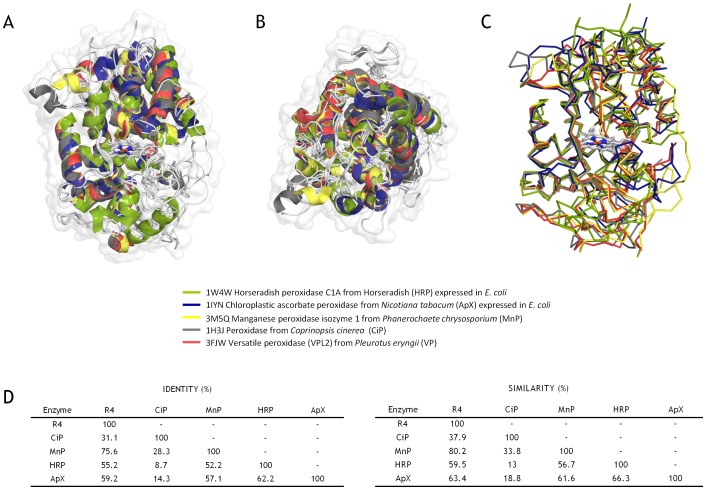
Structural alignment for oxidative stability. (A, B) Front and upper view (cartoon) with the protein surfaces shown in white. (C) Structural alignment in ribbon mode. The prosthetic heme group is highlighted in CPK colors. (D) Identities and similarities for each protein scaffold using the following PDB entries for the analysis: 1W4W, horseradish peroxidase C1A (HRP) expressed in *E. coli* (green); 1IYN, chloroplastic ascorbate peroxidase (ApX) from *Nicotiana tabacum* expressed in *E. coli* (blue); 3M5Q, manganese peroxidase isozyme 1 (MnP) from *Phanerochaete chrysosporium* (yellow); 1H3J, peroxidase from *Coprinopsis cinerea* CiP (gray); and 3FJW, Versatile peroxidase (VPL2) from *Pleurotus eryngii* (red).

**Figure 4:**
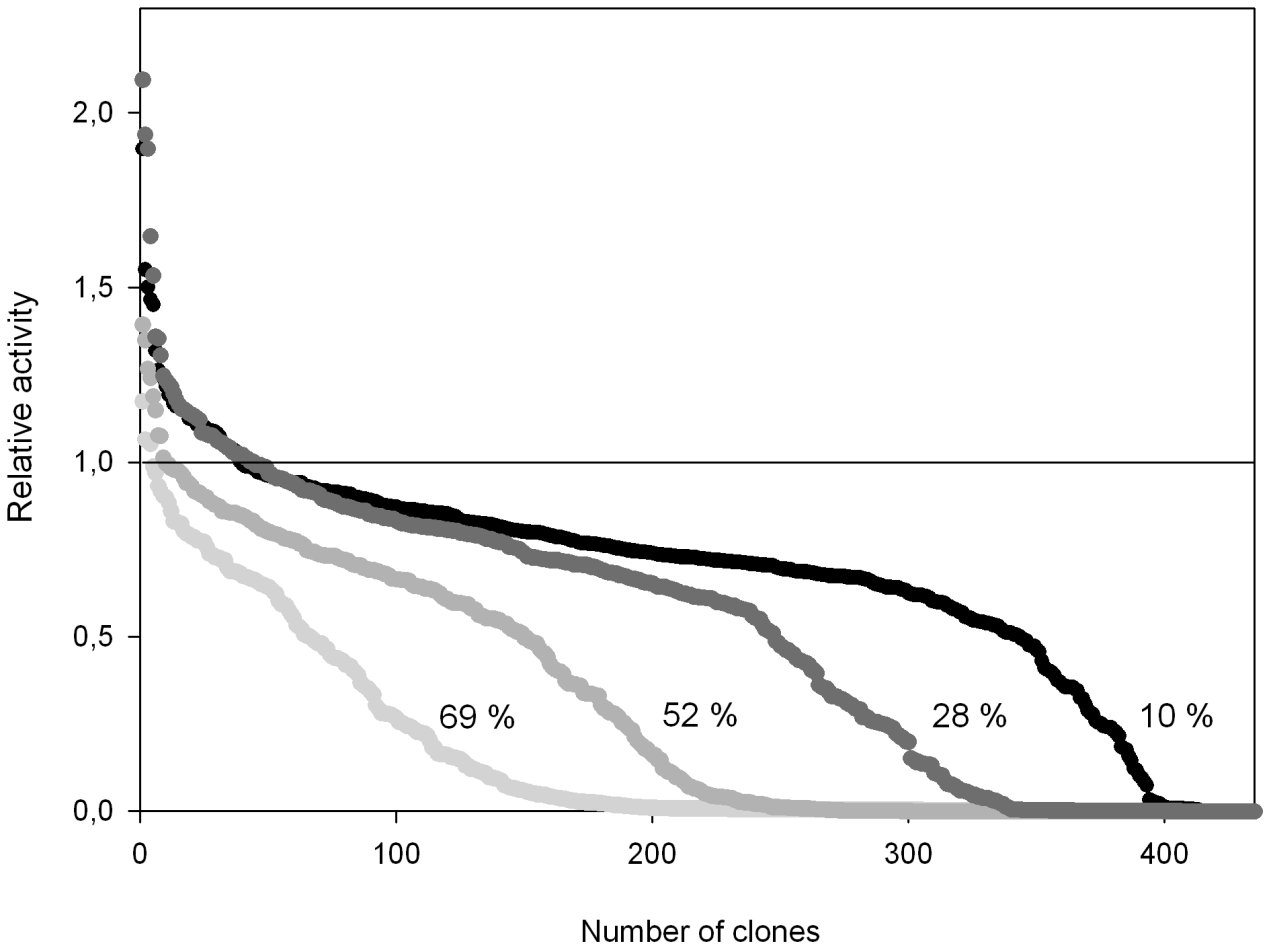
Mutagenic landscapes of the distal His environment (L28-G57) generated using 0.92 ng/µL of DNA template, and 0.01 mM (black), 0.1 mM (dark gray), 0.2 mM (medium gray), 0.4 mM (light gray) of MnCl_2_, respectively. The percentages indicate the number of clones having less than 10% of the parent enzyme's activity; the solid horizontal line indicating the parental activity in the assay.

**Figure 5:**
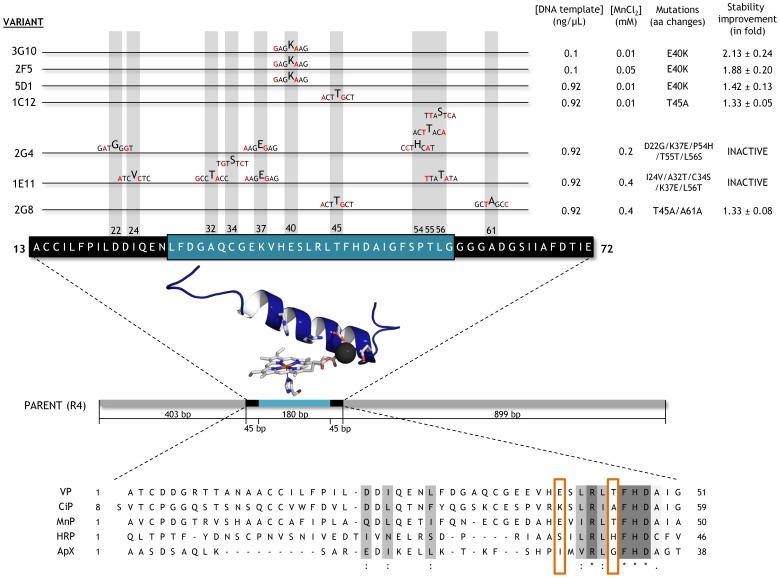
Mutational loads, PCR conditions and selected variants used for MORPHING of the distal His environment. Point mutations are highlighted in red and the overhangs in black. Improvements in oxidative stability are indicated as the fold increase with respect to the parental type at a [H_2_O_2_]/[Enzyme] ratio of 3,000∶1. Sequence alignments with stable peroxidase scaffolds are depicted, framing the mutations at positions 40 and 45 for VP and 53 and 48 for CiP, respectively. The MSA (multiple sequence alignment) was generated with T-coffee software: http://www.igs.cnrs-mrs.fr/Tcoffee/tcoffee_cgi/index.cgi.

To identify what are potentially the most H_2_O_2_-sensitive regions of the VP, we performed a multiple structural alignment using high- and medium-redox potential peroxidases with improved oxidative stability [39], [41]–[43], (**Figure 3**). After careful examination of the model, three different regions (of 26, 30 and 69 amino acids each, excluding the recombination areas) in the vicinity of the heme group were targeted for random mutagenesis and recombination, (**Figures S1, S2**). The first region subjected to MORPHING was the distal His environment (L28-G57) that contains the H_2_O_2_ binding site within a helix that is highly conserved in all high-redox potential peroxidases. The second target region was the proximal His environment (L149-A174) located on the opposite side to the distal His, in the surroundings of the heme domain. The third region was the Met environment (I199-L268), containing three of the five putative oxidizable Met in the VP-R4 variant. Several mutational loads were assayed for each region to construct independent mutant libraries and then explore them for oxidative stability. Mutational loads were adjusted by modifying the PCR conditions in order to introduce 1 to 5 mutations per segment (including the crossover areas). The frequencies of mutation were estimated from different landscapes of mutant libraries (500 clones each), calculating the number of clones with <10% of the parental enzyme activity, and they were further verified by DNA sequencing of a random sample of mutants including active and non-active variants (**Figure 4**). Overall, an average value of T_s_ of 65% (A↔G, 52%; T↔C, 13%) and T_v_ of 35% (T↔A, 17.4%; A↔C, 8.8%; G↔C, 8.8%) was observed in the libraries under study (*i.e.* T_s_/T_v_ ratio∼1.8). Mutational frequencies of 0.5 nucleotide changes/100 bp were obtained with T_s_ G→A when [MnCl_2_]≤0.1 mM, regardless of the DNA template concentration. In segments as short as 30 residues long (*e.g.*, the distal His environment: L28-G57), we obtained high mutational loads at [MnCl_2_]>0.1 mM (frequencies of ∼3 mutations/100 bp). Among the high mutational variants, the 1E11 (2T_s_, 3T_v_) and 2G4 (3T_s_, 2T_v_) mutants incorporated 5 mutations each that inactivated the protein due to the highly conserved nature of this region (**Figure 5**).

**Figure 6:**
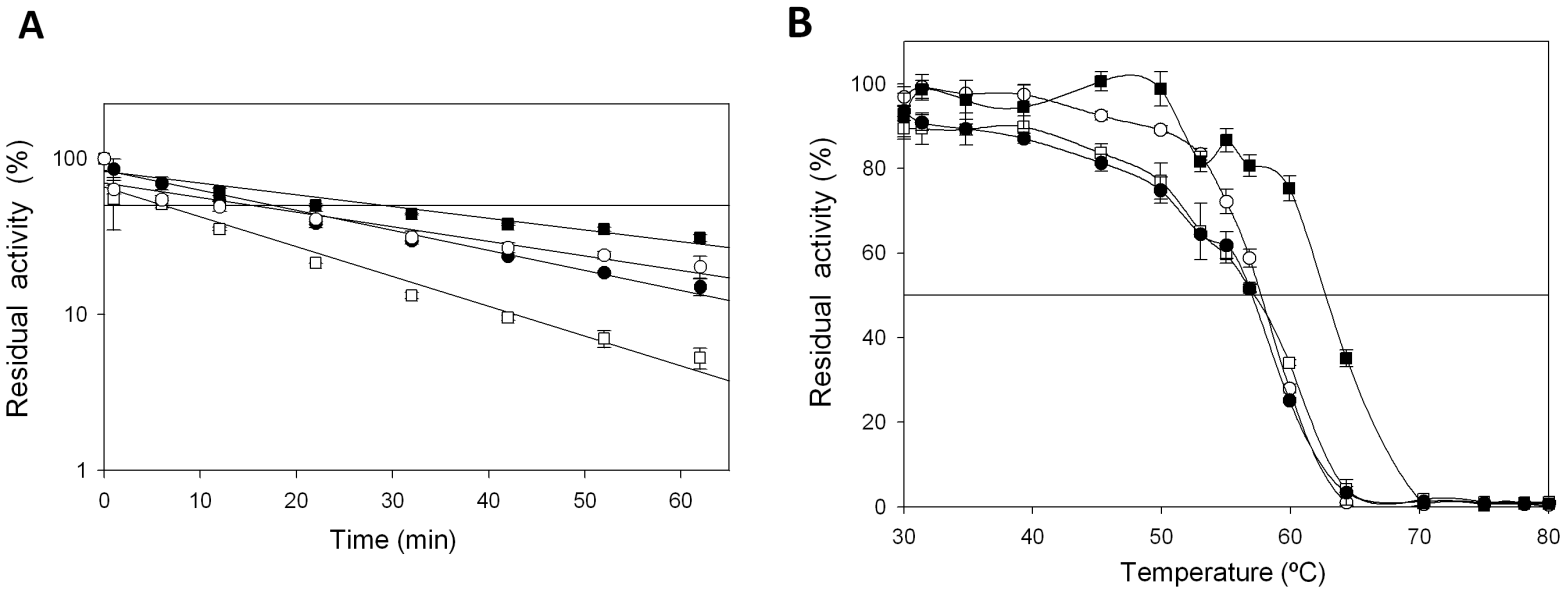
(A) Apparent *t*_1/2_ *vs* H_2_O_2_ for the R4 parental type, 5A9 mutant (P141A), 1C12 mutant (T45A) and 3G10 mutant (E40K) in the presence of 3,000 equivalents of H_2_O_2_. Horizontal line indicates 50% of residual activity. (B) T_50_ profiles (kinetic thermostability) of VP variants. White squares, R4 parental type; white circle, 5A9 mutant (P141A); black squares, 3G10 mutant (E40K); black circles, 1C12 mutant (T45A). Each point represents the mean and standard deviation of three independent experiments.

After the initial screening and three consecutive re-screenings, several mutants with an improved increment in apparent half-life (Δ*t*_1/2_ *vs* H_2_O_2_) were identified. We found two beneficial mutations at the L28-G57 segment. The 3G10 variant showed a 2.1-fold improvement in stability *vs* H_2_O_2_, with a significant Δ*t*_1/2_ *vs* H_2_O_2_ of 18 min with respect to the parental type and a 5.5°C increase in the T_50_ (the temperature at which the enzyme retains 50% of its activity after a 10 min incubation, **Figure 6**). Only one mutation, _GAG_E40K_AAG_, was found in 3G10 and the same mutation was introduced in 2F5 and 5D1 variants. This mutational redundancy highlights the role of this specific alteration in generating oxidative stability in VP and significantly, the highly stable CiP contains a Lys residue at the same position [39], **Figure 5**. In VP, E40 is one of three acidic residues that form the Mn^2+^ binding site (**Figure 2**) and it is plausible that closing some of the protein inlets involved in the generation of free radicals may be beneficial for VP stability, albeit at the cost of compromising this catalytic site. The _ACT_T45A_GCT_ mutation was discovered in both the 1C12 and 2G8 mutants, the latter of which also contained the silent _GCT_A61A_GCC_ mutation (**Figure 5**). While the T45A mutation did not alter thermostability (T_50_ = 59°C), it conferred a 1.3-fold improvement in stability and it was associated with a Δt_1/2_ *vs* H_2_O_2_ of ∼10 min with respect to the parental type (**Figure 6**). The same amino acid (V53A according to CiP numbering) was introduced into the evolved CiP although without improving thermostability, **Figure 5** [39]. It is likely that the only effect of introducing an Ala at this position relates with the accessibility of H_2_O_2_ to the internal protein structure. The _CCT_P141A_GCT_ mutation in the 5A9 variant arose in the L149-A174 segment (proximal His environment). Situated at the heme entrance, Pro141 is a highly conserved residue in all fungal peroxidases. However, the P141A mutation resulted in a 1.4-fold improvement in oxidative stability, producing a Δ*t*_1/2_ *vs* H_2_O_2_ of ∼5 min with respect to the parental type and no change in thermostability (**Figure 6**). P141A is located in a strictly conserved region and according to our model, the substitution of Ala by Pro may widen the heme channel making easier the traffic of oxidized species and therefore limiting their harmful residence time in the inner protein structure. To further study the possible synergies between beneficial mutations E40K, T45A and P141A, a triple variant was constructed by site-directed mutagenesis doubling the Δ*t*_1/2_ *vs* H_2_O_2_ to 45 min (data not shown). This mutant was the departure point to engineer an oxidative stable VP variant by successive rounds of *in vitro* evolution (unpublished material).

No mutants were discovered in the I199-L268 segment (the Met environment), suggesting that the three oxidizable methionines in this area are not involved in the oxidative stabilization of VP. This result was corroborated by subjecting Met262 and Met265 to combinatorial saturation mutagenesis and screening. No improved variants were identified in this way and ∼95% of the clones were inactive in this mutagenic landscape, indicating that these residues are very sensitive and do not tolerate changes (**Figure S3**). Similarly, Met152 in the proximal His environment was not mutated.

In this first enzyme case study, MORPHING effectively identified new structural determinants, such as the P141A mutation, that are important for oxidative stability. Significantly, the identification of the E40K and T45A mutations was in good agreement with previous findings [39], thereby validating this approach. Although each mutated segment was analyzed independently by constructing small high-quality libraries with different mutational loads, it is also feasible that different combinations of mutated segments can be prepared using the yeast's recombination machinery.

### Enhancing functional UPO expression

**Figure 7:**
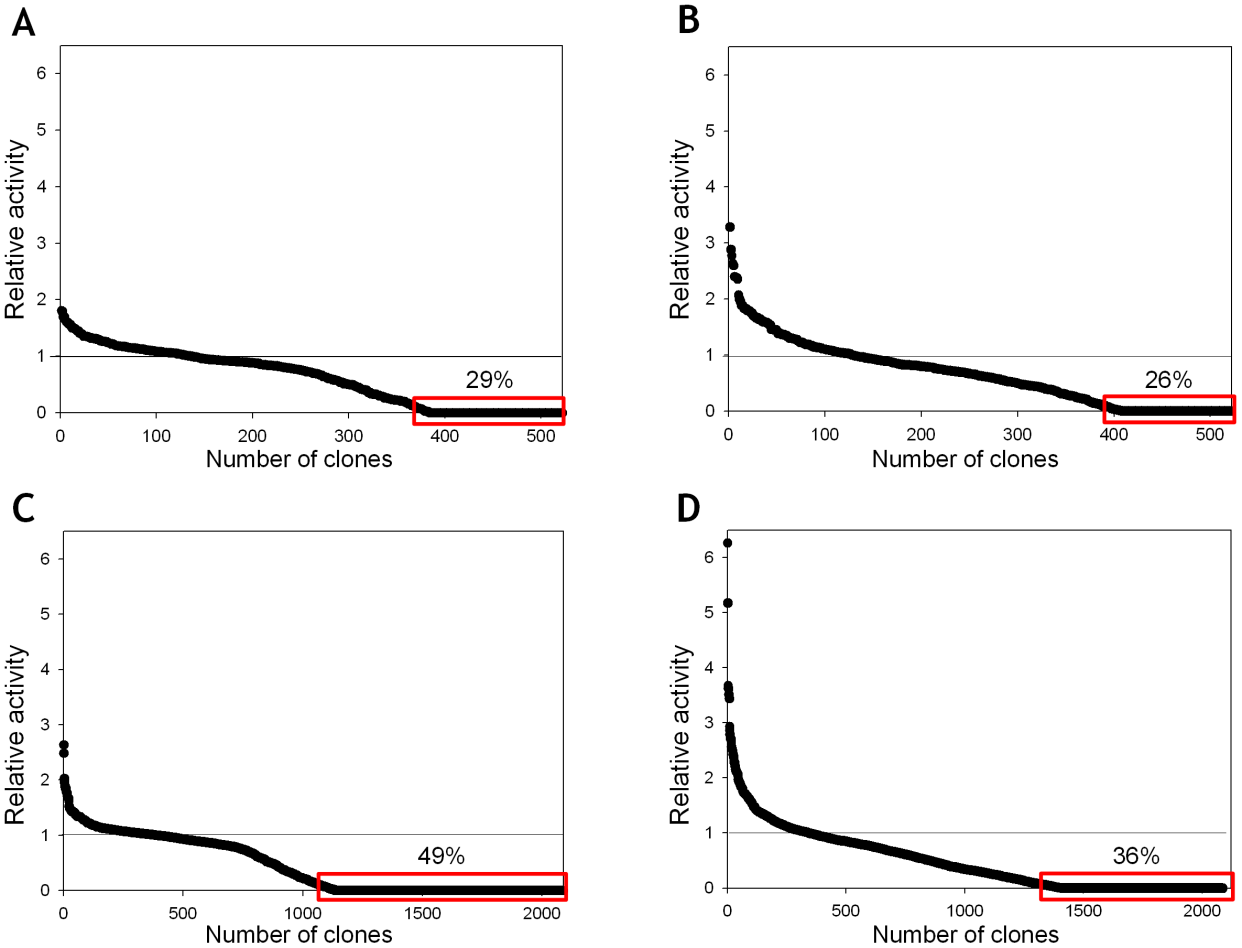
Mutagenic landscapes for MORPHING of the signal peptide of UPO using ABTS (A) and NBD (B) in colorimetric assays. Directed evolution landscapes of the whole UPO gene obtained with ABTS (C) and NBD (D) assays. The activity of the clones is plotted in descending order. The solid horizontal line indicates the activity of the parental type in the assay.

MORPHING was also used to assess whether the secretion levels of an unspecific peroxygenase (UPO) could be enhanced in *S. cerevisiae*. UPO (EC 1.11.2.1) is a new, potentially ligninolytic, peroxidase that has attracted much research attention due to its versatility and applicability in a variety of synthetic processes [35], [44]. Its peroxygenative (oxygen-transfer) activity is of particular importance as UPO can behave as a self-sufficient monooxygenase to mediate regio- and enantio-selective oxyfunctionalizations that are essential for organic synthesis. Among the array of oxygen transfer reactions catalyzed by UPO are brominations, sulphoxidations, N-oxidations, aromatic peroxygenations, alkyl hydroxylations, double bond epoxidations and ether cleavages. Like many other ligninolytic oxidoreductases, UPO is not readily expressed in heterologous hosts so that it can be tailored by directed evolution and therefore, we recently addressed this problem by subjecting the whole UPO gene to several rounds of random mutagenesis, recombination and screening in *S. cerevisiae* (unpublished data). To further enhance the secretion of this protein, we used MORPHING to independently evolve the 43 amino acid UPO signal peptide in order to enrich the signal leader in beneficial mutations without altering the biochemical properties of the enzyme. To reliably compare the two directed evolution strategies, we exposed the signal peptide alone and the entire UPO gene (including its signal leader) to one round of directed evolution in two parallel experiments. Mutational rates for the leader library and the full-gene library were 0.5 and 0.1 nucleotide changes/100 bp, respectively. Both libraries were screened with the help of an ad-hoc dual-colorimetric assay to estimate the enzymes peroxidase (with ABTS) and peroxygenase (with NBD) activities. The mutagenic landscape generated by MORPHING revealed an increased tolerance to mutations in the signal peptide than in the whole UPO gene. Indeed, we found that when random mutagenesis of the leader and the whole UPO gene was compared, 30% and 49% of clones had <10% of parental enzyme activity on ABTS as a substrate, respectively (**Figure 7**). These results are consistent with the fact that mutations in the leader sequence only affect secretion, whereas those in the whole UPO gene may also compromise the catalytic properties. While no variants carrying mutations in the leader were identified in the whole UPO gene after one round of evolution, several independent but almost consecutive mutations were observed in three independent beneficial variants after MORPHING of the leader sequence (_TTC_F12Y_TAC_, _GCG_A14V_GTG_ and _AGG_R15G_GGG_). Each of these mutations individually enhanced secretion by ∼20% with respect to the parental type. Although MORPHING was useful to unmask these beneficial mutations for secretion, the eukaryotic machinery of *S. cerevisiae* was unable to join these positions by homologous recombination due to their proximity. Therefore, we constructed a signal peptide containing the full set of these mutations by conventional site-directed mutagenesis looking for a synergic effect in secretion levels. The evolved signal peptide also included the beneficial mutation _GCC_A21D_GAC_ (discovered in the earlier stages of evolution). Finally, the signal peptide of native UPO was replaced by the evolved signal peptide F12Y-A14V-R15G-A21D (see Materials and Methods for details), resulting in a 27-fold increase in total secretion compared to the native UPO fused to its original leader (∼2±0.11 ABTS U/L and 54±5.7 ABTS U/L, respectively). F12Y-A14V-R15G-A21D mutations are located at the hydrophobic core of the leader and they may exert a beneficial effect on secretion by promoting a more suited interaction with the signal recognition particle during translocation to the endoplasmic reticulum.

This second case study demonstrates that the use of our focused mutagenesis method to direct the evolution of signal leaders is a suitable approach to promote heterologous functional expression of complex eukaryotic genes in yeast. Targeting mutational loads to leader sequences is a simple means of detecting mutations that are beneficial for secretion and that can be subsequently combined in a single signal peptide to generate potential synergies along the *S. cerevisiae* secretory pathway.

### Conclusions

The random domain mutagenesis/recombination method presented here is a reliable *one-pot* approach for the construction of focused mutant libraries of eukaryotic genes in *S. cerevisiae*. In general terms, MORPHING allows the researcher to focus exclusively on the random introduction of mutations and their recombination in restricted region/s, while protecting critical domains from mutagenesis. The selection of the target regions to be evolved is as important as the mutational loads chosen for each mutant library, which can be easily varied to enrich the target segments in beneficial mutations.

The two case studies presented here validate the versatility of our method by tackling two distinct problems. While MORPHING proved useful to explore limited targeted regions, allowing us to identify several structural determinants of H_2_O_2_ inhibition in VP that could be applied to other high redox-potential fungal peroxidases, it also effectively decoupled secretion and catalytic activity for functional UPO expression. This approach can also be used to explore other complex problems, such as to alter substrate specificity or enantio-selectivity by subjecting several segments of the same gene to random mutagenesis, promoting their *in vivo* assembly in one transformation step. Indeed, this strategy is currently being used by our group to evolve a fungal aryl alcohol oxidase for the selective oxidation of different alcohols. Apart from structure-function relationship studies, MORPHING can be also useful when structural information is absent, *e.g.*, for the evolution of leader peptides for secretion, for the modification of promoters, or in the evolution of unknown regions of biochemical relevance that have been revealed by conventional directed evolution.

Additional advantages of this method include the reduction of the sequence space to be explored (good results can be achieved with small libraries of 400–500 clones), the conservation of certain catalytic properties while improving other traits, and the discovery of new structural/catalytic determinants that can be further optimized using focused saturation mutagenesis. The combination of MORPHING with classical directed evolution and semi-rational approaches, or with neutral genetic drift, may lead to the development of new adaptive pathways to engineer more robust eukaryotic enzymes in yeast [8], [13], [14].

## Supporting Information

Figure S1 **VP MORPHING.** Three different regions of VP were targeted for random mutagenesis and recombination (L28-G57, L149-A174, and I199-L268). The VP gene is shown in blue, the α-factor prepro-leader to promote secretion in yeast in red and the shuttle vector in green. The areas of crossover between the fragments are represented by crosses. The overlapping areas between segments were created by superimposing PCR reactions in defined regions (see also **Figure S2** and **Table S1**).
(TIF)

Figure S2 **Selected areas of VP subjected to MORPHING: proximal His environment (red), Met environment (yellow), and distal His environment (blue).** The heme domain is depicted in stick mode and CPK colors. The amino acids delimiting mutated regions and the most relevant residues are highlighted (proximal and distal histidines; Mn^2+^ binding pocket with manganese represented as a grey sphere).
(TIF)

Figure S3 **Combinatorial saturation mutagenesis landscapes at positions 262 and 265 of VP.** Clone activity is plotted in descending order. The solid horizontal line indicates the activity of the parental type in the assay.
(TIF)

Table S1 **Oligos used for VP and UPO MORPHING.** The lengths of the PCR products and the overlapping areas are shown. NNS and SNN indicate NN(G/C) and (G/C)NN codons for the saturation mutagenesis libraries. CSM, combinatorial saturation mutagenesis; UPOsp, UPO signal peptide; IvAM, *In vivo* Assembly of Mutant libraries for the directed evolution of the whole UPO gene; epPCR, error-prone PCR; HF-PCR, high-fidelity PCR.
(TIF)

## References

[1] Bershtein S, Tawfik DS (2008) Advances in laboratory evolution of enzymes. Curr Opin Chem Biol 12: 151–158. 18284924 10.1016/j.cbpa.2008.01.027

[2] Bloom JD, Arnold FH (2009) In the light of directed evolution: pathways of adaptive protein evolution. Proc Natl Acad Sci U S A 106 Suppl 1: 9995–10000. 19528653 10.1073/pnas.0901522106 PMC2702793

[3] Cobb RE, Si T, Zhao H (2012) Directed evolution: an evolving and enabling synthetic biology tool. Curr Opin Chem Biol 16: 285–291. 22673064 10.1016/j.cbpa.2012.05.186 PMC3424333

[4] Romero PA, Arnold FH (2009) Exploring protein fitness landscapes by directed evolution. Nat Rev Mol Cell Biol 10: 866–876. 19935669 10.1038/nrm2805 PMC2997618

[5] Tracewell CA, Arnold FH (2009) Directed enzyme evolution: climbing fitness peaks one amino acid at a time. Curr Opin Chem Biol 13: 3–9. 19249235 10.1016/j.cbpa.2009.01.017 PMC2703427

[6] Lutz S (2010) Beyond directed evolution-semi-rational protein engineering and design. Curr Opin Biotechnol 21: 734–743. 20869867 10.1016/j.copbio.2010.08.011 PMC2982887

[7] Wong TS, Roccatano D, Schwaneberg U (2007) Steering directed protein evolution: strategies to manage combinatorial complexity of mutant libraries. Environ Microbiol 9: 2645–2659. 17922750 10.1111/j.1462-2920.2007.01411.x

[8] Gupta RD, Tawfik DS (2008) Directed enzyme evolution via small and effective neutral drift libraries. Nat Methods 5: 939–942. 18931667 10.1038/nmeth.1262

[9] Peisajovich SG, Tawfik DS (2007) Protein engineers turned evolutionists. Nat Methods 4: 991–994. 18049465 10.1038/nmeth1207-991

[10] Bloom JD, Romero PA, Lu Z, Arnold FH (2007) Neutral genetic drift can alter promiscuous protein functions, potentially aiding functional evolution. Biol Direct 2: 17. 17598905 10.1186/1745-6150-2-17 PMC1914045

[11] Bloom JD, Lu Z, Chen D, Raval A, Venturelli OS, et al (2007) Evolution favors protein mutational robustness in sufficiently large populations. BMC Biol 5: 29. 17640347 10.1186/1741-7007-5-29 PMC1995189

[12] Voloshchuk N, Montclare JK (2010) Incorporation of unnatural amino acids for synthetic biology. Mol Biosyst 6: 65–80. 20024068 10.1039/b909200p

[13] Dalby PA (2011) Strategy and success for the directed evolution of enzymes. Curr Opin Struct Biol 21: 473–480. 21684150 10.1016/j.sbi.2011.05.003

[14] Goldsmith M, Tawfik DS (2013) Enzyme engineering by targeted libraries. Methods Enzymol 523: 257–283. 23422434 10.1016/B978-0-12-394292-0.00012-6

[15] Shivange AV, Marienhagen J, Mundhada H, Schenk A, Schwaneberg U (2009) Advances in generating functional diversity for directed protein evolution. Curr Opin Chem Biol 13: 19–25. 19261539 10.1016/j.cbpa.2009.01.019

[16] Reetz MT, Bocola M, Carballeira JD, Zha D, Vogel A (2005) Expanding the range of substrate acceptance of enzymes: combinatorial active-site saturation test. Angew Chem Int Ed Engl 44: 4192–4196. 15929154 10.1002/anie.200500767

[17] Reetz MT, Carballeira JD (2007) Iterative saturation mutagenesis (ISM) for rapid directed evolution of functional enzymes. Nat Protoc 2: 891–903. 17446890 10.1038/nprot.2007.72

[18] Alcolombri U, Elias M, Tawfik DS (2011) Directed evolution of sulfotransferases and paraoxonases by ancestral libraries. J Mol Biol 411: 837–853. 21723874 10.1016/j.jmb.2011.06.037

[19] Hidalgo A, Schliessmann A, Molina R, Hermoso J, Bornscheuer UT (2008) A one-pot, simple methodology for cassette randomisation and recombination for focused directed evolution. Protein Eng Des Sel 21: 567–576. 18559369 10.1093/protein/gzn034

[20] Herman A, Tawfik DS (2007) Incorporating Synthetic Oligonucleotides via Gene Reassembly (ISOR): a versatile tool for generating targeted libraries. Protein Eng Des Sel 20: 219–226. 17483523 10.1093/protein/gzm014

[21] Hong KK, Nielsen J (2012) Metabolic engineering of *Saccharomyces cerevisiae*: a key cell factory platform for future biorefineries. Cell Mol Life Sci 69: 2671–2690. 22388689 10.1007/s00018-012-0945-1 PMC11115109

[22] Krivoruchko A, Siewers V, Nielsen J (2011) Opportunities for yeast metabolic engineering: Lessons from synthetic biology. Biotechnol J 6: 262–276. 21328545 10.1002/biot.201000308

[23] Nevoigt E (2008) Progress in metabolic engineering of *Saccharomyces cerevisiae* . Microbiol Mol Biol Rev 72: 379–412. 18772282 10.1128/MMBR.00025-07 PMC2546860

[24] Da Silva NA, Srikrishnan S (2012) Introduction and expression of genes for metabolic engineering applications in *Saccharomyces cerevisiae* . FEMS Yeast Res 12: 197–214. 22129153 10.1111/j.1567-1364.2011.00769.x

[25] Alcalde M (2010) Mutagenesis protocols in *Saccharomyces cerevisiae* by In Vivo Overlap Extension. Methods Mol Biol 634: 3–14. 20676972 10.1007/978-1-60761-652-8_1

[26] Ostrov N, Wingler LM, Cornish W (2013) Gene assembly and combinatorial libraries in *S. cerevisiae* via reiterative recombination. Methods Mol Biol 978: 187–203. 23423898 10.1007/978-1-62703-293-3_14

[27] Zumarraga M, Camarero S, Shleev S, Martinez-Arias A, Ballesteros A, et al (2008) Altering the laccase functionality by in vivo assembly of mutant libraries with different mutational spectra. Proteins 71: 250–260. 17932916 10.1002/prot.21699

[28] Shao Z, Zhao H, Zhao H (2009) DNA assembler, an in vivo genetic method for rapid construction of biochemical pathways. Nucleic Acids Res 37: e16. 19074487 10.1093/nar/gkn991 PMC2632897

[29] Pompon D, Nicolas A (1989) Protein engineering by cDNA recombination in yeasts: shuffling of mammalian cytochrome P-450 functions. Gene 15 83 (1) 15–24. 10.1016/0378-1119(89)90399-5 2687113

[30] Wingler LM, Cornish VW (2011) Reiterative Recombination for the *in vivo* assembly of libraries of multigene pathways. Proc Natl Acad Sci 13: 15135–40. 10.1073/pnas.1100507108 PMC3174608 21876185

[31] Gonzalez-Perez D, Garcia-Ruiz E, Alcalde M (2012) *Saccharomyces cerevisiae* in directed evolution: An efficient tool to improve enzymes. Bioeng. Bugs 3: 172–177. 10.4161/bbug.19544 PMC3370936 22572788

[32] Wang T, Ma X, Zhu H, Li A, Du G, Chen J (2012) Available methods for assembling expression cassettes for synthetic biology. Appl Microbiol Biotechnol 93: 1853–1863. 22311648 10.1007/s00253-012-3920-8

[33] Finney-Manchester SP, Maheshri N (2013) Harnessing mutagenic homologous recombination for targeted mutagenesis in vivo by TaGTEAM. Nucleic Acids Res 41: e99. 23470991 10.1093/nar/gkt150 PMC3643572

[34] Ruiz-Dueñas FJ, Morales M, Garcia E, Miki Y, Martinez MJ, et al (2009) Substrate oxidation sites in versatile peroxidase and other basidiomycete peroxidases. J. Exp. Bot 60: 441–452. 10.1093/jxb/ern261 18987391

[35] Hofrichter M, Ullrich R (2006) Heme-thiolate haloperoxidases: versatile biocatalysts with biotechnological and environmental significance. Appl Microbiol Biotechnol 71: 276–288. 16628447 10.1007/s00253-006-0417-3

[36] Garcia-Ruiz E, Gonzalez-Perez D, Ruiz-Duenas FJ, Martinez AT, Alcalde M (2012) Directed evolution of a temperature-, peroxide- and alkaline pH-tolerant versatile peroxidase. Biochem J 441: 487–498. 21980920 10.1042/BJ20111199

[37] Pecyna MJ, Ullrich R, Bittner B, Clemens A, Scheibner K, et al (2009) Molecular characterization of aromatic peroxygenase from *Agrocybe aegerita* . Appl Microbiol Biotechnol 84: 885–897. 19434406 10.1007/s00253-009-2000-1

[38] Wong TS, Zhurina D, Schwaneberg U (2006) The diversity challenge in directed protein evolution. Comb Chem High Throughput Screen 9 (4) 271–88. 16724918 10.2174/138620706776843192

[39] Cherry JR, Lamsa MH, Schneider P, Vind J, Svendsen A, et al (1999) Directed evolution of a fungal peroxidase. Nat Biotechnol 17: 379–384. 10207888 10.1038/7939

[40] Valderrama B, Ayala M, Vazquez-Duhalt R (2002) Suicide inactivation of peroxidases and the challenge of engineering more robust enzymes. Chem Biol 9: 555–565. 12031662 10.1016/s1074-5521(02)00149-7

[41] Miyazaki-Imamura C, Ohira K, Kitagawa R, Nakano H, Yamane T, Takahashi H (2003) Improvement of H_2_O_2_ stability of manganese peroxidase by combinatorial mutagenesis and high-throughput screening using in vitro expression with protein disulfide isomerase. Protein Eng 16: 423–428. 12874375 10.1093/protein/gzg054

[42] Ryan BJ, O'Fagain C (2007) Effects of single mutations on the stability of horseradish peroxidase to hydrogen peroxide. Biochimie 89: 1029–1032. 17482746 10.1016/j.biochi.2007.03.013

[43] Kitajima S, Kitamura M, Koja N (2008) Triple mutation of Cys26, Trp35, and Cys126 in stromal ascorbate peroxidase confers H_2_O_2_ tolerance comparable to that of the cytosolic isoform. Biochem Biophys Res Commun 372: 918–923. 18539135 10.1016/j.bbrc.2008.05.160

[44] Hofrichter M, Ullrich R, Pecyna MJ, Liers C, Lundell T (2010) New and classic families of secreted fungal heme peroxidases. Appl Microbiol Biotechnol 87: 871–897. 20495915 10.1007/s00253-010-2633-0

