# Supplementary figures and images for: Mutagenic Organized Recombination Process by Homologous In Vivo Grouping (MORPHING) for Directed Enzyme Evolution
Source: PLoS One. 2014 Mar 10;9(3):e90919. doi: 10.1371/journal.pone.0090919 (PMC3948698; doi:10.1371/journal.pone.0090919)

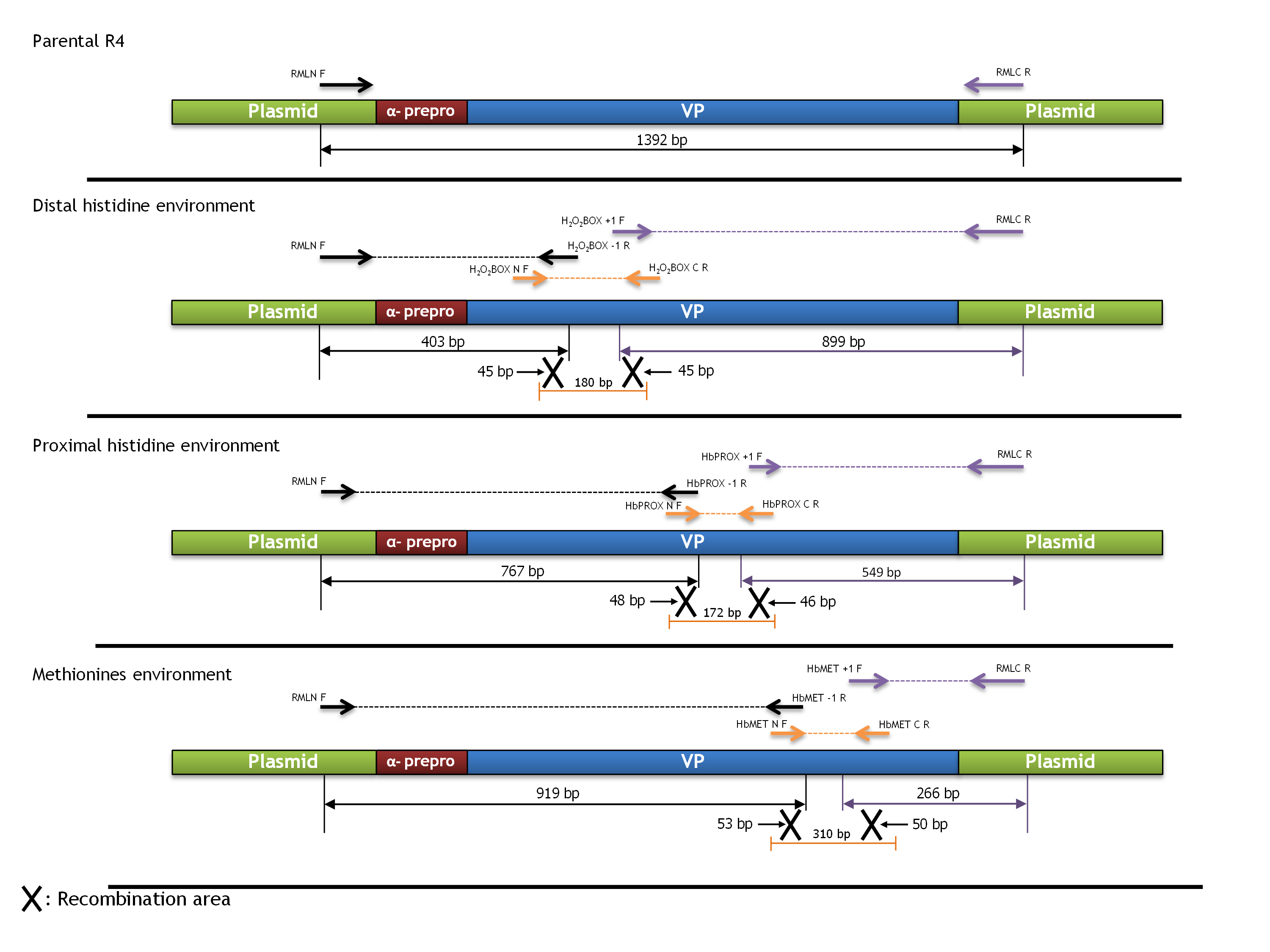

Supplement: Figure S1 — VP MORPHING. Three different regions of VP were targeted for random mutagenesis and recombination (L28-G57, L149-A174, and I199-L268). The VP gene is shown in blue, the α-factor prepro-leader to promote secretion in yeast in red and the shuttle vector in green. The areas of crossover between the fragments are represented by crosses. The overlapping areas between segments were created by superimposing PCR reactions in defined regions (see also Figure S2 and Table S1). (TIF) [file pone.0090919.s001.tif]

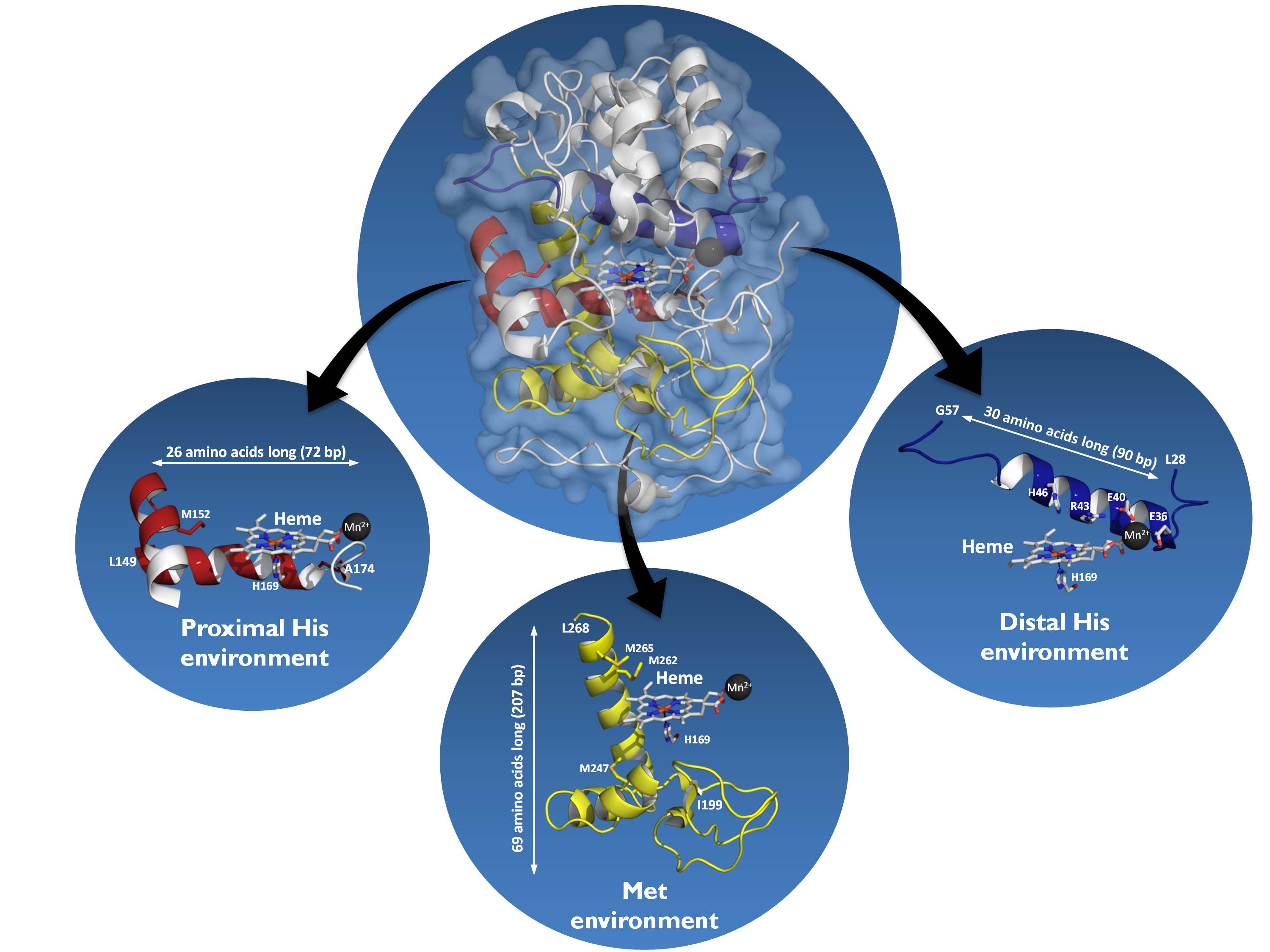

Supplement: Figure S2 — Selected areas of VP subjected to MORPHING: proximal His environment (red), Met environment (yellow), and distal His environment (blue). The heme domain is depicted in stick mode and CPK colors. The amino acids delimiting mutated regions and the most relevant residues are highlighted (proximal and distal histidines; Mn2+ binding pocket with manganese represented as a grey sphere). (TIF) [file pone.0090919.s002.tif]

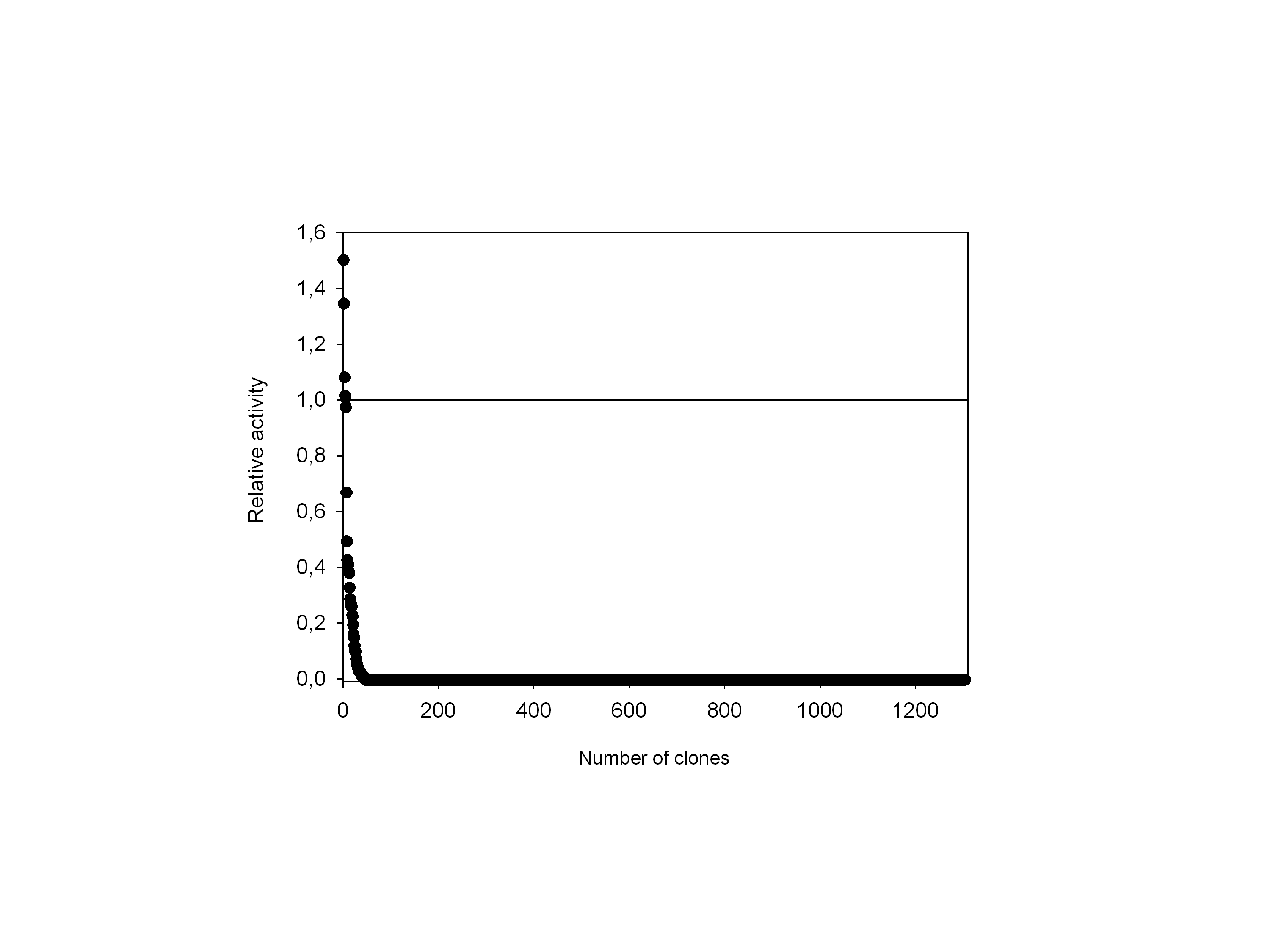

Supplement: Figure S3 — Combinatorial saturation mutagenesis landscapes at positions 262 and 265 of VP. Clone activity is plotted in descending order. The solid horizontal line indicates the activity of the parental type in the assay. (TIF) [file pone.0090919.s003.tif]

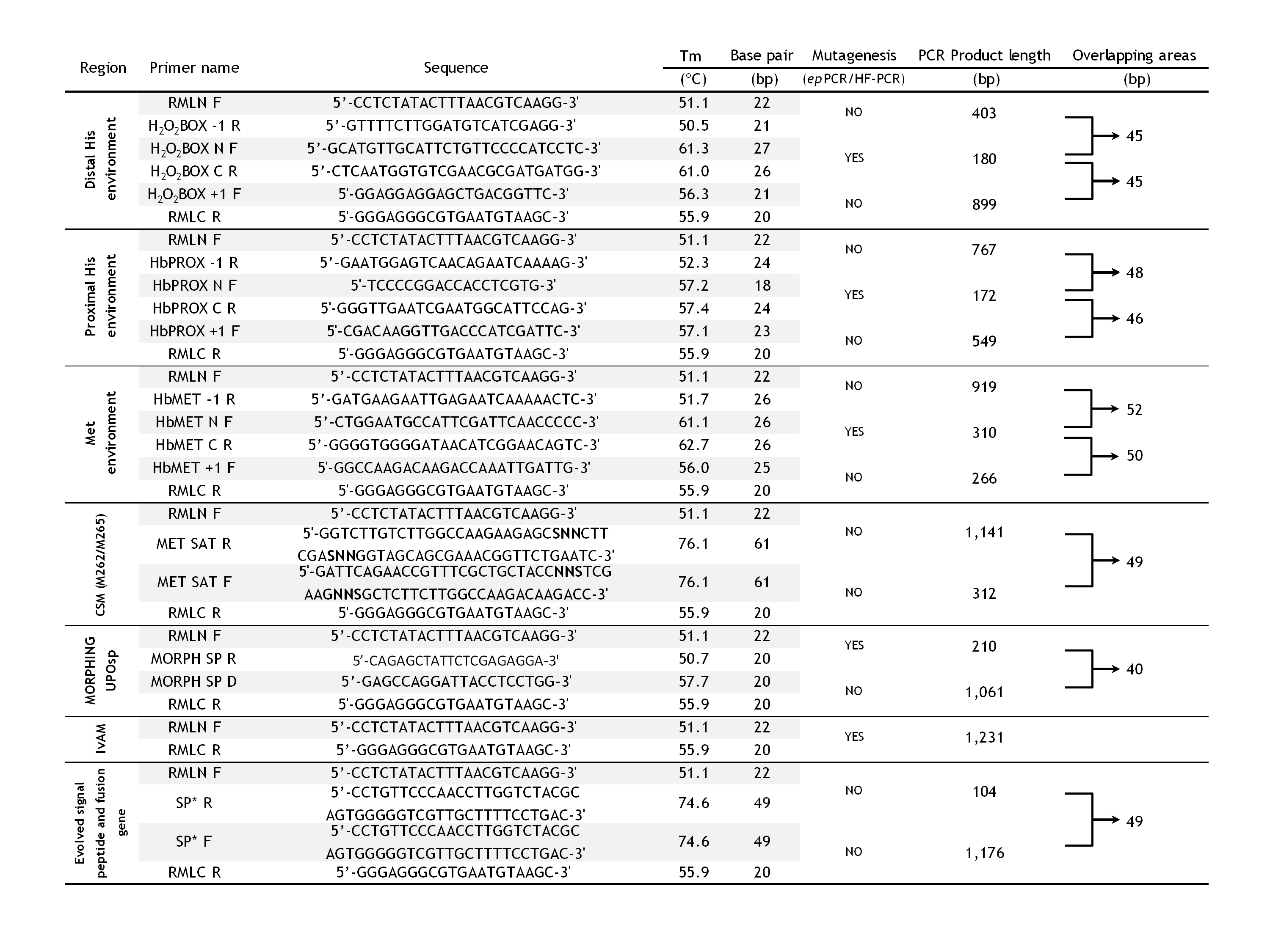

Supplement: Table S1 — Oligos used for VP and UPO MORPHING. The lengths of the PCR products and the overlapping areas are shown. NNS and SNN indicate NN(G/C) and (G/C)NN codons for the saturation mutagenesis libraries. CSM, combinatorial saturation mutagenesis; UPOsp, UPO signal peptide; IvAM, In vivo Assembly of Mutant libraries for the directed evolution of the whole UPO gene; epPCR, error-prone PCR; HF-PCR, high-fidelity PCR. (TIF) [file pone.0090919.s004.tif]
